# Human papillomavirus 16L1-58L2 chimeric virus-like particles elicit durable neutralizing antibody responses against a broad-spectrum of human papillomavirus types

**DOI:** 10.18632/oncotarget.19327

**Published:** 2017-07-18

**Authors:** Xue Chen, Hongyang Liu, Zhirong Wang, Shuo Wang, Ting Zhang, Meili Hu, Liang Qiao, Xuemei Xu

**Affiliations:** ^1^ Department of Biophysics and Structural Biology, Institute of Basic Medical Sciences, Chinese Academy of Medical Sciences, School of Basic Medicine, Peking Union Medical College, Beijing 100005, China; ^2^ Department of Microbiology and Immunology, Stritch School of Medicine, Loyola University Chicago, Maywood, Illinois 60153, USA; ^3^ Biotherapy Center, The First Affiliated Hospital of Zhengzhou University, Zhengzhou, Henan 450052, China; ^4^ Institute of Precision Medicine, Jining Medical University, Jining, Shandong 272067, China

**Keywords:** human papillomavirus, HPV58 L2, chimeric virus-like particle, vaccine, cross-neutralizing antibodies

## Abstract

The neutralizing antibodies elicited by human papillomavirus (HPV) major capsid protein L1 virus-like particle (VLP)-based vaccines are largely type-specific. An HPV vaccine inducing cross-neutralizing antibodies broadly will be cost-effective and of great value. To this end, we constructed HPV16L1-58L2 chimeric VLP (cVLP) by displaying HPV58 L2 aa.16-37 on the DE surface region of HPV16 L1. We found that vaccination with the HPV16L1-58L2 cVLP formulated with alum plus monophosphoryl lipid A (Alum-MPL) adjuvant elicited robust neutralizing antibodies in both mice and rabbits against all tested HPV types including HPV16/31/33/35/52/58 (genus *α9*), HPV18/39/45/59/68 (genus *α7*), HPV6/11 (genus *α10*), HPV2/27/57 (genus *α4*), and HPV5 (genus *β1*). Importantly, the cross-neutralizing antibody response was maintained at least 82 weeks in mice or 42 weeks in rabbits, and complete protection against HPV58 was observed at week 85 in mice. Our data demonstrate that HPV16L1-58L2 cVLP is an excellent pan-HPV vaccine candidate.

## INTRODUCTION

Over 200 human papillomavirus (HPV) types, which are members of five genera (*α, β, γ, μ, v*) [1, 2], are responsible for approximately 5% of all human cancers and substantial precancerous and benign lesions [3, 4]. Persistent infection with high-risk mucosal HPV (HPV 16/18/31/33/35/39/45/51/52/56/58/59/68/73/82 etc) is the etiological cause of nearly all cervical cancer, which is the third most common cancer in women worldwide, and a proportion of other anogenital (vaginal, vulvar, penile and anal) and oropharyngeal cancers [5, 6]. Low-risk mucosal types HPV6/11 (*α10*) are main causative agents for condyloma acuminatum and recurrent respiratory papillomatosis [7, 8]. Cutaneous HPV2/27/57 (*α4*) and HPV1 (*μ1*) are common types in cutaneous warts [9, 10]. Infection with HPV5/8 (*β1*) is associated with squamous cancer in individuals suffering from epidermodysplasia verruciformis [11].

Three available HPV L1 VLP-based prophylactic vaccines, bivalent Cervarix (contains HPV16/18 VLPs), quadrivalent Gardasil (contains HPV16/18/6/11 VLPs), and nonavalent Gardasil-9 (contains HPV16/18/31/33/45/52/58/6/11 VLPs), have shown to provide protection against vaccine types [12–16]. There is also evidence of limited cross-protection against HPV31/33/45 with Cervarix or against HPV31 with Gardasil [17–19], but the strength and duration of cross-neutralizing antibody responses are lower and shorter than that of the vaccine types [19–22]. While the nonavalent vaccine provides broader protection against oncogenic HPV infections and infection-related precancerous lesions, it still does not cover the cutaneous HPV types. Moreover, continuing to add more and more types of VLPs in a vaccine also raises the complexity and cost of production. High cost remains the primary challenge to global implementation of HPV vaccines, especially in the developing countries where nearly 90% of cervical cancer deaths occur [23, 24].

An alternative approach to fill in the gap between cross-protection and cost is focused on developing a vaccine using the minor capsid protein L2. Vaccination with the N-terminus of L2 induces cross-neutralizing antibodies, but the antibody titers against the homologous HPVs are lower than that induced by L1 VLPs [25–27]. Approaches to boost the immunogenicity of L2 include multimeric display of epitopes on surface region of VLPs from papillomavirus [28–30], adeno-associated virus [31], tobacco mosaic virus [32], or on surface region of recombinant bacteriophage [33, 34], and delivery of epitopes with FcγR-targeting scaffolds [35, 36], bacterial thioredoxin or flagellin scaffold [37, 38]. Immunization of KLH-conjugated HPV16 L2 aa.17-36 peptide elicited cross-neutralizing antibodies [39]. The homologous region of L2 aa.17-36 peptide (usually termed RG1 epitope) derived from HPV16, 33 or 45 has been inserted into the DE loop of HPV16 or HPV18 L1VLP to create L1-L2 chimeric VLP vaccines, which have been demonstrated to induce various titers of cross-neutralizing antibodies with different spectrum [29, 30, 40].

HPV16 is the most common oncogenic type worldwide and accounts for approximately 60% of invasive cervical cancer (ICC) [41, 42]. However, the prevalence of other types varies in different regions. For example, HPV58 and 52 are more prevalent oncogenic types in Eastern Asia (ranked third and fourth) than other regions [41–45].

**Figure 1:**
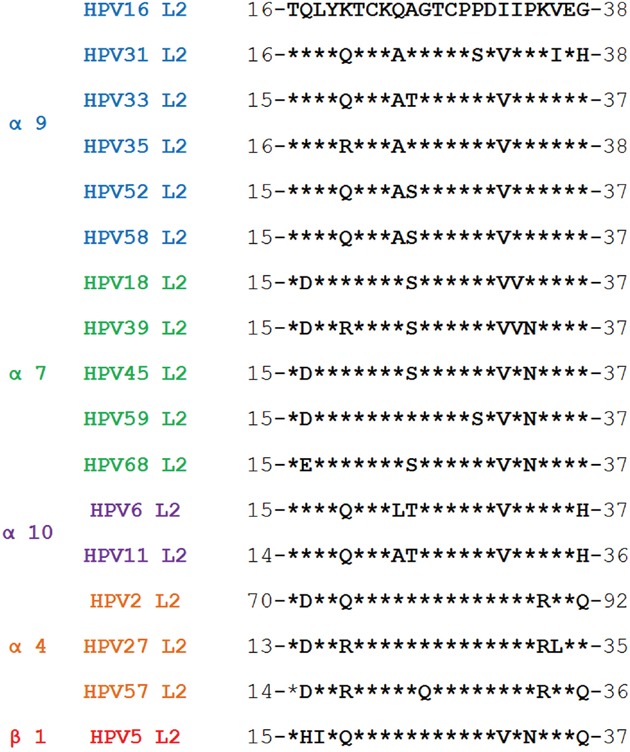
Homologous analysis of HPV58 L2 aa.15-37 peptide and comparable sequences from different HPV types

Because HPV58 is highly prevalent in China, we have examined the potential of HPV58 L2 aa.15-37 (Figure 1, 100% identity with HPV52) to induce cross-neutralizing antibodies and have generated HPV16L1-58L2 cVLP vaccine by inserting HPV58 L2 aa.16-37 into the DE surface loop of HPV16 L1 VLP. We have found that the chimeric VLP vaccine could induce long-term cross-neutralizing immune responses against a broad-spectrum of HPV types.

## RESULTS

### HPV58 L2 aa.15-37 peptide induces broadly neutralizing antibodies in rabbits

The success of HPV16 RG1 peptide in inducing cross-neutralizing antibody responses lead us to test if HPV58 (the third most prevalent high-risk mucosal type in Eastern Asia) and HPV6 (the most prevalent low-risk mucosal type worldwide) RG1 peptides are also able to do so. HPV58 and HPV6 RG1 peptides were synthesized and conjugated with KLH (termed KLH-58RG1 and KLH-6RG1, respectively). Two rabbits per group were immunized four times with KLH-58RG1 or KLH-6RG1 and sera were collected two weeks after the fourth immunization to determine the neutralizing antibody titers against 13 types of HPV pseudoviruses (PsVs).

As shown in Table 1, KLH-58RG1 antisera neutralized all 13 tested HPV PsVs, including HPV58 (mean titer, 2,000), HPV33 (mean titer, 1,000), HPV52/45 (mean titer, 450/450), HPV11 (mean titer, 400), HPV39 (mean titer, 250), HPV18 (mean titer, 225), HPV31/6 (mean titer, 150/150), HPV35 (mean titer, 75), HPV16/5 (mean titer, 50/50) and HPV59 (mean titer, 25). In contrast, KLH-6RG1 antisera neutralized only 10 tested HPV PsVs with very low titers. Given the ability to induce cross-neutralizing antibodies broadly against *α9, α7, α10* and *β1* genera, HPV58 RG1 peptide was chosen to develop chimeric VLPs.

**Table 1:** Neutralizing antibody response of rabbits vaccinated 4 times with KLH-58RG1 or KLH-6RG1

PsVs		Neutralizing antibody titer			
Genus & species	Types	KLH-58RG1		KLH-6RG1	
		1	2	1	2
α9	HPV16	50	50	<25	50
	HPV31	100	200	<25	<25
	HPV33	1600	400	50	100
	HPV35	50	100	<25	50
	HPV52	800	100	<25	<25
	HPV58	3200	800	<25	50
α7	HPV18	50	400	<25	50
	HPV39	100	400	<25	100
	HPV45	100	800	<25	100
	HPV59	25	25	<25	<25
α10	HPV6	100	200	50	200
	HPV11	400	400	<25	25
β1	HPV5	50	50	<25	25

Rabbits (n=2) were subcutaneously primed with 1 mg of KLH-58RG1 or KLH-6RG1 formulated with CFA and boosted with 500 μg of antigens formulated with IFA 3 times. Sera were collected two weeks after the fourth immunization for PsV-based neutralization analysis.

### Generation of HPV16L1-58L2 cVLPs

**Figure 2:**
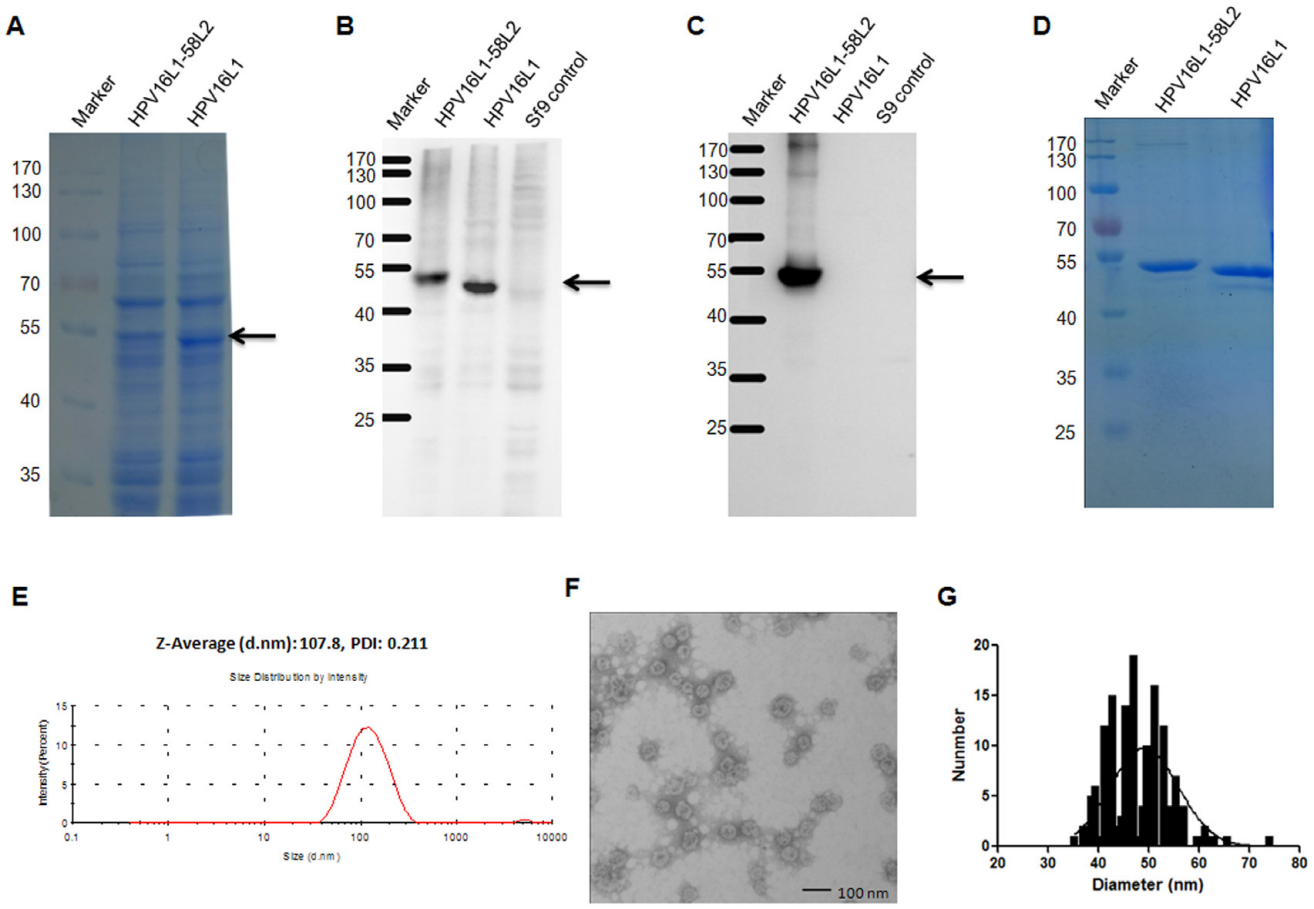
Analysis of the HPV16L1-58L2 chimeric protein Lysis of Sf9 cells expressing HPV16L1-58L2 or HPV16 L1 protein was analyzed by SDS-PAGE with coomassie blue staining **(A)**, and Western blot with Camvir-1 MAb **(B)** or anti-HPV58 L2 aa.15-37 polyclonal rabbit serum **(C)**. Uninfected Sf9 cell lysate is used as control in B and C. The purity of HPV16L1-58L2 protein was determined by SDS-PAGE with coomassie blue staining **(D)**. Pure HPV16L1-58L2 protein was analyzed by DLS **(E)** and TEM **(F)**. The diameter distribution of HPV16L1-58L2 cVLP was shown by frequency histogram **(G)**.

To potentially enhance and broaden cross-neutralizing responses induced by the HPV58 RG1 peptide, we engineered an HPV16L1-58L2 chimeric protein by displaying HPV58 L2 aa.16-37 on the DE loop of HPV16 L1 and expressed it in baculovirus expression system. The HPV16L1-58L2 protein was highly expressed similar to HPV16L1 protein as analyzed by SDS-PAGE with coomassie blue staining (Figure 2A). The HPV16L1-58L2 protein was confirmed by Western blot with Camvir-1 monoclonal antibody or polyclonal rabbit serum raised against HPV58 L2 aa.15-37 showing a strong migrating band at 50-55 kDa (Figure 2B, 2C). After purification by ultracentrifugation, HPV16L1-58L2 protein was tested by SDS-PAGE with coomassie blue staining and a strong band was observed when 5 μg of each protein was loaded (Figure 2D). The purity was about 90% as tested by HCP ELISA. Dynamic light scattering (DLS) analyses of purified HPV16L1-58L2 protein showed the uniform hydrodynamic diameter distribution with a mean polydispersity index of 0.211 and an average hydrodynamic diameter of 107.8 nm (Figure 2E). Further transmission electron microscopy (TEM) analysis showed that HPV16L1-58L2 protein was assembled into full-size VLPs (average diameter, 49 nm) with the unimodal distribution of particle diameter (Figure 2F, 2G).

### HPV16L1-58L2 cVLPs induced broadly cross-neutralizing antibody responses in mice

**Figure 3:**
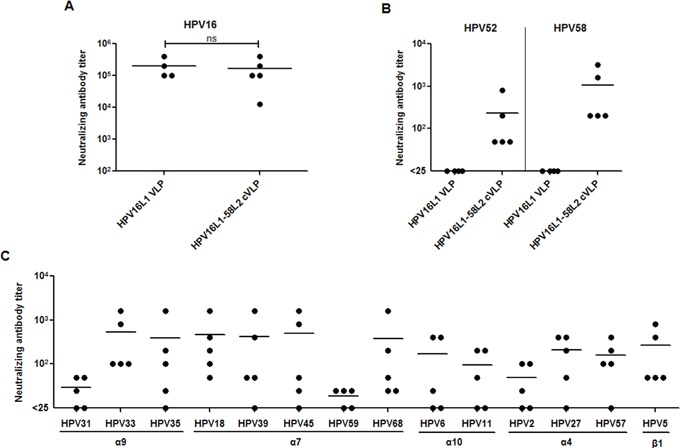
HPV16L1-58L2 cVLPs induced broadly cross-neutralizing antibody response in mice Mice (n=4/5) were immunized subcutaneously at weeks 0, 4, 7, 10 with 10 μg of HPV16 L1 VLP or HPV16L1-58L2 cVLP adjuvanted with Alum-MPL. Sera were collected at week 12 and analyzed for neutralization against vaccine types **(A, B)**, and non-vaccine types **(C)**. The horizontal bars represent the geometric mean antibody titers. The statistically significant differences (using two-tailed, unpaired *t*-test) were indicated by: ns, *P*>0.05.

To test the ability of HPV16L1-58L2 cVLPs to induce neutralizing antibodies, sera from mice (n=5) immunized with Alum-MPL adjuvanted HPV16L1-58L2 cVLPs were analyzed by standard PsV-based neutralization assay (PBNA). Immunization with HPV16L1-58L2 cVLPs induced potent neutralizing antibody against HPV16 with mean titer of 166,400, which was comparable with that by HPV16 L1 VLPs (*P>*0.05, Figure 3A). HPV16L1-58L2 cVLPs also induced robust neutralizing antibodies against HPV58 (mean titer, 1,080) and a little weaker against HPV52 (mean titer, 230), whereas HPV16 L1 VLP antisera had no detectable neutralizing antibodies against HPV52/58 (Figure 3B). Therefore, these data indicate that insertion of HPV58 L2 aa.16-37 does not interfere the exposure of major neutralizing epitopes of HPV16 L1 VLPs and the HPV58 RG1 peptide displayed on the chimeric VLPs is highly immunogenic. We next determined the cross-neutralizing antibody responses against non-vaccine types. As shown in Figure 3C, we detected cross-neutralizing antibodies against high-risk mucosal HPV31/33/35 (*α9*) (mean titers, 25/540/385), HPV18/39/45/59/68 (*α7*) (mean titers, 470/420/495/15/380), low-risk mucosal HPV6/11 (*α10*) (mean titers, 165/90), as well as cutaneous HPV2/27/57 (*α4*) (mean titers, 45/210/160) and HPV5 (*β1*) (mean titer, 270). However, sera from mice immunized with HPV16 L1 VLPs had very weak cross-neutralizing activity against HPV31 and non-detectable against other HPV types tested (not shown). Taken together, HPV16L1-58L2 cVLPs formulates with human-applicable Alum-MPL adjuvant were able to induce cross-neutralizing antibody responses to common high/low-risk mucosal and cutaneous HPV types in mice.

### HPV16L1-58L2 cVLPs induced cross-neutralizing antibody responses against diverse HPV types in rabbits

**Figure 4:**
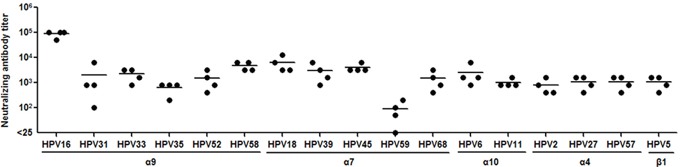
HPV16L1-58L2 cVLPs induced broadly cross-neutralizing antibody response in rabbits NZW rabbits (n=4) were immunized at weeks 0, 4, 7, 10 with 50 μg of HPV16L1-58L2 cVLPs formulated with Alum-MPL. Sera were collected at week 12 and analyzed for cross-neutralization against 17 HPV types with PBNA. The horizontal bars represent the geometric mean antibody titers.

We next asked whether the cross-neutralizing antibody response by HPV16L1-58L2 cVLPs in mice is translatable to large animal rabbits to further evaluate the potential clinical application of the vaccine. Four rabbits were vaccinated with HPV16L1-58L2 cVLPs in Alum-MPL and sera were drawn 2 weeks after the fourth immunization to test the neutralizing activities against 17 HPV types (11 high-risk mucosal, 2 low-risk mucosal and 4 cutaneous types) by PBNA. As shown in Figure 4, similar to what we found in mice, HPV16L1-58L2 cVLPs also elicited cross-neutralizing antibodies against all tested 17 HPV types in rabbits, including HPV16 (mean titer, 89,600), HPV18 (mean titer, 6,400), HPV58 (mean titer, 4,800), HPV45 (mean titer, 4,000), HPV39 (mean titer, 3,000), HPV6 (mean titer, 2,600), HPV33 (mean titer, 2,200), HPV31 (mean titer, 2,025), HPV52/68 (mean titer, 1,500/1,500), HPV27/57/5 (mean titer, 1,100/1,100/1,100), HPV11 (mean titer, 1,000), HPV2 (mean titer, 800), HPV35 (mean titer, 650) and HPV59 (mean titer, 88).

### HPV16L1-58L2 cVLPs induced long-term neutralizing antibody responses in both mice and rabbits

**Figure 5:**
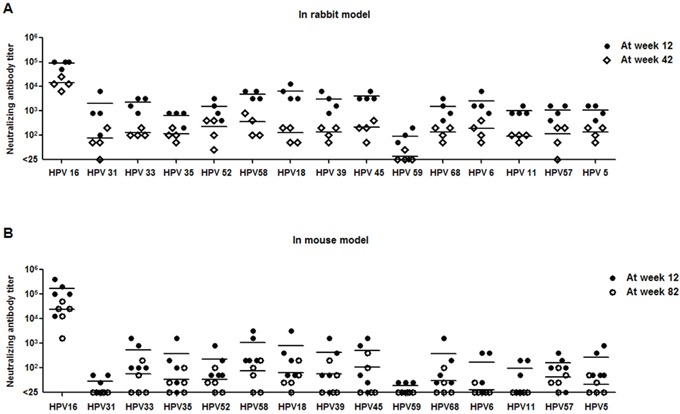
HPV16L1-58L2 cVLPs induced long-lasting neutralizing antibody responses HPV16L1-58L2 cVLPs immune sera were collected at weeks 12 and 42 from rabbits **(A)** or at weeks 12 and 82 from mice **(B)**, and tested with PBNA. HPV16/31/33/35/52/58/18/39/45/59/68/6/11/57/5 PsVs for rabbit (n=4) and mouse (n=5) sera analysis. The horizontal bars represent the geometric mean antibody titers.

To further explore the long-term duration of neutralizing antibodies induced by Alum-MPL adjuvanted HPV16L1-58L2 cVLP vaccine, rabbits (n=4) and mice (n=5) were immunized at weeks 0, 4, 7 and 10. Rabbit sera were collected at weeks 12 and 42, and mouse sera were collected at weeks12 and 82 to determine the cross-neutralizing antibody levels. Although the titers were 6-51 folds lower than those at week 12, antisera from HPV16L1-58L2 cVLP immunized rabbits at week 42 were still able to neutralize all 15 tested HPV PsVs (Figure 5A), including HPV16 (mean titer, 14,400), HPV58 (mean titer, 350), HPV52 (mean titer, 231), HPV45 (mean titer, 213), HPV6 (mean titer, 188), HPV39/68/5 (mean titer, 138/138/138), HPV18/33 (mean titer, 125/125), HPV35/57 (mean titer, 113/113), HPV11 (mean titer, 88), HPV31 (mean titer, 75) and HPV59 (mean titer, 6). To examine the duration of neutralizing antibody responses longer than 42 weeks, we determined the neutralizing antibody levels in the antisera collected from mice at week 82. We observed strong neutralizing antibody response against HPV16 (mean titer, 23,360) and detectable neutralizing antibodies against HPV45 (mean titer, 100), HPV58 (mean titer, 70), HPV18 (mean titer, 60), HPV52/33/39 (mean titer, 50/50/50), HPV57 (mean titer, 40), HPV35 (mean titer, 30), HPV68 (mean titer, 25), HPV5 (mean titer, 15) and HPV6 (mean titer, 5) (Figure 5B). Therefore, our data suggest that HPV16L1-58L2 cVLPs can induce long-term neutralizing antibody responses in both mice and rabbits.

### HPV16L1-58L2 cVLPs induced long-term protection in mice

**Figure 6:**
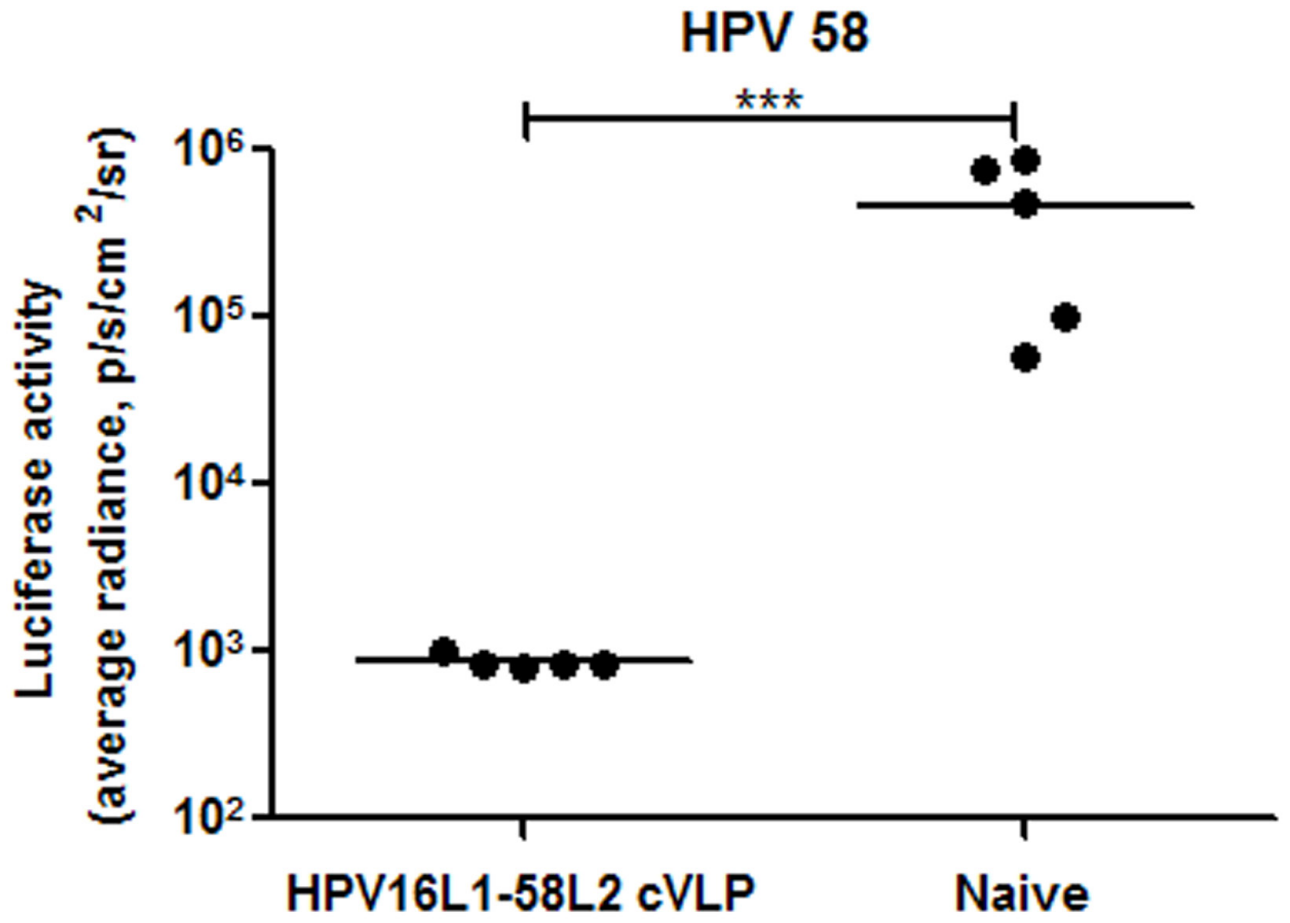
HPV16L1-58L2 cVLP induced long-term protection in mice Mice (n=5) were immunized subcutaneously at weeks 0, 4, 7, 10 with 10 μg of HPV16L1-58L2 cVLP adjuvanted with Alum-MPL. At week 85, mice were intravaginally challenged with HPV58 pseudovirus. Luciferase signals were acquired for 10 min with Xenogen IVIS Spectrum 48 h after PsV infection. Infection is measured as bioluminescence. The statistically significant differences (using one-tailed unpaired *t*-test) were indicated by: ***, *P*<0.001.

As shown in Figure 5, the cross-neutralizing antibody response induced by HPV16L1-58L2 cVLPs was maintained at least 42 weeks in rabbits and 82 weeks in mice. The antibody titer threshold for protection has not been clearly identified, but antibody titers are more than 100-fold lower than the minimum titer detectable by standard PBNA are sufficient to confer protection against PsV genital infections in mouse model [46]. To evaluate the long-term protective response induced by HPV16L1-58L2 cVLPs, the immunized mice were vaginal challenged with HPV58 PsV at week 85. Although 2 of 5 neutralizing antibody titers against HPV58 were not detectable at week 82, complete protection was still conferred at week 85 (Figure 6).

## DISCUSSION

HPV L1 VLPs are highly immunogenic but induce mainly type-specific neutralizing antibodies. The recently approved Gardasil-9 contains HPV L1 VLPs from 7 types of high-risk HPVs and 2 types of low-risk HPVs. Thus, an HPV vaccine that can be produced cost-effectively and covers a broader spectrum of HPV types than Gardasil-9 is desired. In this study, we used HPV16 L1 VLP as the backbone to display HPV58 L2 aa.16-37 into HPV16 L1 DE loop. We found that the chimeric VLPs induced broadly neutralizing antibodies against over 17 HPV types. Thus, we believe that it is a promising candidate for a new generation of HPV vaccine.

Although RG1 sequences from different HPVs are relatively conserved (Figure 1), it remains unclear whether the RG1 peptide from other HPVs (except for HPV16) can induce strong and broad-spectrum neutralizing antibodies. Jagu et al. [26] showed that sera from mice immunized with a synthetic tandem repeat of L2 aa.17-36 derived from HPV58 and other 21 HPV types neutralized HPV16/18/45, but not HPV58/6. Tumban et al. [33] showed that mice vaccinated with a mixture containing 8 recombinant PP7 bacteriophages (a single-strand RNA bacteriophage of *Pseudomonas aeroginosa*) each displaying one type of HPV L2 aa.17-31 (including HPV58), provided only partial *in vivo* protection against HPV58, and the neutralizing activity of the antisera was not known. To our knowledge, the immunogenicity of HPV58 RG1 peptide to elicit cross-neutralizing antibody responses is not well studied. Here we found that immunization of KLH-58RG1 peptide could induce cross-neutralizing antibody responses against all 13 tested HPV types with relatively high titers whereas KLH-6RG1 antisera neutralized only 10 HPVs with very low titers. These data indicated that the immunogenicity of any HPV type RG1 peptide is difficult to be empirically predicted.

As the immunogenicity of HPV58 RG1 is relatively high, we inserted HPV58 L2 aa.16-37 into the DE surface loop of HPV16 L1 VLP to generate HPV16L1-58L2 chimeric protein. The protein was highly expressed with good stability and efficiently self-assembled into VLPs (Figure 2), and induced potent broad-spectrum neutralizing antibody responses (Figures 3, 4). Contrarily, the HPV16L1-6L2 chimeric protein, which constructed by inserting HPV6 L2 aa.17-38 into the DE surface loop of HPV16 L1 VLP, was shown to be unstable, and induced much lower levels of antibody response even against HPV6 (not shown). There are three amino acid differences (86% homology) between HPV58 L2 aa.16-37 and HPV6 L2 aa.17-38, which may influence the interaction between the residues of inserted peptide and L1 backbone, and result in the differences in stability and immunogenicity of the two chimeric proteins. Therefore, it is important to optimize L2 peptides with good immunogenicity and select L1-L2 cVLPs with high stability for screening vaccine candidates.

Schellenbacher et al. [40] reported a chimeric 16L1-16RG1 VLP, which was generated by inserting HPV16 L2 aa.17-36 peptide into the DE loop of HPV16 L1 between aa.136 and aa.137 without introduction of amino acid between the peptide and adjacent residues of L1 backbone, and used rabbit model to investigate the cross-neutralizing antibody responses against 33 HPV types, include 20 high-risk HPV PsV types, 5 low-risk HPVs (4 types of PsVs, 1 type of native virion) and 8 cutaneous HPVs (5 types of PsVs, 3 types of native virion), and found that the antisera (n=6) neutralized 17 high-risk HPVs, the mean titers from high to low are HPV16 (titer, 7,000), HPV31 (titer, 1,833), HPV18/35/73 (titer, 550/550/550), HPV34 (titer, 542), HPV58 (titer, 388), HPV45 (titer, 358), HPV70 (titer, 217), HPV68 (titer, 183), HPV39 (titer, 120), HPV26 (titer, 92), HPV33 (titer, 63), HPV52 (titer, 38), HPV69 (titer, 17), HPV59/51 (titer, 4). Only the two most potent sera were tested for cross-neutralization against low-risk and cutaneous HPVs and found to neutralize 3 low-risk and 3 cutaneous HPV types of PsVs, the mean titers of HPV6/32 is 75/75, HPV11 is 50, HPV3 is 1,000, HPV76 is 100 and HPV5 is 75. In this study, HPV16L1-58L2 cVLP rabbit antisera (n=4) neutralized all 17 tested of HPV types, including 11 high-risk, 2 low-risk mucosal and 4 cutaneous types. The mean titers of the rabbit antisera from high to low are HPV16 (titer, 89,600), HPV18 (titer, 6,400), HPV58 (titer, 4,800), HPV45 (titer, 4,000), HPV39 (titer, 3,000), HPV6 (titer, 2,600), HPV33 (titer, 2,200), HPV31 (titer, 2,025), HPV52/68 (titer, 1,500/1,500), HPV27/57/5 (titer, 1,100/1,100/1,100), HPV11 (titer, 1,000), HPV2 (titer, 800), HPV35 (titer, 650), HPV59 (titer, 88). Thus, HPV16L1-58L2 cVLP induced broad-spectrum neutralizing antibody responses, and is another promising pan-HPV vaccine candidate. We noticed that, the cross-neutralizing antibody responses induced by 16RG1 peptide varied when delivered with different scaffold proteins [35, 47], cross-neutralizing antibody titers in descending order of thioredoxin-L2 (20-38) sera were against HPV16/18/58/45/31 [47]; while that of a modified Fc-16RG1 fusion protein sera were against HPV16/18/45/5/52/58/6/11 [35]. We speculated that the linear amino acid of a RG1 epitope may exhibit different secondary structures when delivered with different scaffolds, and result in different antibody responses. Thus in this study, we modified the L1 amino acids adjacent to the N- and C-terminus of inserted peptide with glycine-proline and proline respectively, which may provide structural flexibility and facilitate the inserted epitope to form proper secondary structure.

The long lasting cross-neutralizing antibody responses are essential for HPV protection since HPV vaccines are mostly given to preadolescents, the protection should sustain a decade, at least from preadolescence through midlife. Previous studies have indicated that *in vivo* protection induced by either L1 VLP- or L2-based vaccine is mediated by neutralizing antibodies [48–52]. Schellenbacher et al. [40] determined the duration of neutralizing antibodies induced by 16L1-16RG1 VLP to 8 HPV PsVs and found that they could detect neutralizing antibodies to HPV16/18/31, but not to other 5 types, at week 52. Our data showed that HPV16L1-58L2 cVLP induced long lasting neutralizing antibody response, the neutralizing antibodies against 15 HPVs that were maintained at relatively high levels at week 42 in rabbits (Figure 5A). It also induced durable neutralizing antibody response in mice up to week 82 (Figure 5B), the longest neutralizing antibody response among the L2-based vaccines reported so far to our best knowledge.

Although increasing L1 VLP type in multivalent VLP vaccines can broaden the vaccine protection spectrum, it is not cost-effective since adding one VLP type mainly increases one protection type. We believe that formulating multivalent VLP vaccines with the HPV16L1-58L2 cVLP will induce much broader protection spectrum and similar level of HPV16 neutralizing antibody responses comparing to the vaccines formulated with HPV16 L1 VLP, since the HPV16L1-58L2 cVLP induce similar high titers of HPV16 neutralizing antibodies as HPV16 L1 VLP, and various titers of cross-neutralizing antibodies against a broad spectrum of HPVs.

In summary, our study demonstrate that the HPV16L1-58L2 cVLP is an attractive pan-HPV vaccine candidate to induce strong and durable cross-neutralizing antibodies against a broad-spectrum HPV types, representing a novel candidate for HPV vaccines.

## MATERIALS AND METHODS

### Ethics statement

All animal work was done in accordance with the guidelines of the Institutional Animal Care and Use Committee of the Institute of Laboratory Animal Science, Chinese Academy of Medical Sciences, and all experimental protocols were approved by the Institutional Animal Care and Use Committee.

### Synthetic peptide

Synthetic peptides that mimic HPV58 L2 aa.15-37 (100% identity with HPV52) or HPV6 L2 aa.16-39 were synthesized by GL Biochem LTD (Shanghai). Each peptide was conjugated to keyhole limpethemocyanin (KLH) via the N-(3-Dimethylaminopropyl)-N’-ethylcarbonate linker.

### Generation of HPV16L1-58L2 chimeric protein

Our previous work showed that the optimized *HPV16L1* delta C31 gene (accession number: GU556964) expressed HPV16 L1 VLP at high level in baculovirus expression system [53]. In this study *HPV58 L2 aa.16-37* gene sequence was genetically engineered into the DE loop of the above *HPV16 L1* between nt.408 and nt.412 (aa.136 and aa.138) with three point mutations in the flanking sequence (Y135G, A136P, N138P) by PCR strategy. The *HPV16L1-58L2* construct was subcloned into pFastBac1 vector and expressed in the Bac-to-Bac baculovirus expression system according to the manufacturer's instructions (Invitrogen).

HPV16L1-58L2 chimeric proteins were purified by CsCl ultracentrifugation as described previously [53] with minor modifications. Briefly, the supernatant of cell lysate was purified by ultracentrifugation on 27% CsCl (w/w)-PBS density gradients for 20 h, then dialyzed the interest fraction in 0.5 M NaCl PBS at 4°C for 2-3 h and ultracentrifugation on 5% sucrose (w/w)/60% CsCl (w/w)-0.5 M NaCl PBS for 2.5 h and then on 27% CsCl (w/w)-0.5 M NaCl PBS density gradients for 20 h. VLPs were dialyzed against PBS at 4°C for 3 days. Cell lysates or purified proteins were analyzed by SDS-PAGE with coomassie blue staining or Western blot with Camvir-1 (MILLIPORE, MAB885) or polyclonal rabbit serum raised against HPV58 L2 aa.15-37.

### Dynamic light scattering (DLS) and transmission electron microscopy (TEM)

DLS measurements were performed on a Malvern Zetasizer Nano ZS (Malvern). Samples were filtered through a 0.45 μm filter followed by equilibration to 25°C. The hydrodynamic size was recorded as Z-average hydrodynamic diameter. All data reported here are the averages of three measurements of the same sample.

For TEM analysis, purified proteins were adsorbed on a carbon-coated grid for 1 minute, rinsed with distilled water, negatively stained with 1% uranyl acetate for 3 minutes and examined with a TEM-1400 electron microscope operating at 80 kV with a magnification of 80,000×.

### Animal immunizations

New Zealand white (NZW) rabbits and BALB/c mice were purchased from the Institute of Laboratory Animal Science, Chinese Academy of Medical Sciences, and kept in the animal facility of the Institute of Basic Medical Sciences, Chinese Academy of Medical Sciences.

NZW rabbits (n=2) were immunized subcutaneously on days 0, 14, 28 and 42 with 1 mg of synthetic HPV58 L2 aa.15-37 or HPV6 L2 aa.16-39 coupled to KLH (termed KLH-58RG1 or KLH-6RG1, respectively) in complete Freund's adjuvant (CFA) with the primary dose and 500 μg of antigens in incomplete Freund's adjuvant (IFA) with boost doses. Sera were collected two weeks after the fourth immunization and stored at −20°C.

Female BALB/c mice (n=5) or NZW rabbits (n=4) were vaccinated subcutaneously at weeks 0, 4, 7 and 10 with 10 or 50 μg of HPV16L1-58L2 cVLPs formulated in Alum-MPL, respectively. HPV16 L1 VLPs adjuvanted with Alum-MPL was used as a control. Sera were collected at weeks 12, 42 or 82 and stored at −20°C.

### HPV pseudovirus preparation

PsVs of HPV2/5/6/11/16/18/27/31/33/35/39/45/ 52/57/58/59/68 with encapsidated reporter plasmid pfwB which encoding green fluorescence protein (GFP) or pLucf which encoding both luciferase and GFP were produced in 293TT cells as previously described [35].

### Standard PsV-based neutralization assay (PBNA)

Standard PBNA was performed as described previously [35]. Every sample was analyzed in duplicate. Statistical significance was determined by two-tailed, unpaired t-test in Graphpad Prism 5. *P* values <0.05 were considered to be statistically significant.

### Murine vaginal HPV PsV challenge

Murine vaginal HPV PsV challenge was performed as described previously [35, 36]. Mice were treated with 3 mg of progesterone subcutaneously four days before PsV challenge. The immunized mice were intravaginally pretreated with 50 μl of 4% nonoxynol-9 (Sigma-Aldrich, USA) at six hours prior to PsV challenge. 20 μl of PsV preparation containing 5.0×10^5^ IU of PsVs (encapsidated reporter plasmid pLucf) and 1% carboxymethyl cellulose (Sigma-Aldrich, USA) was intravaginally instilled using a positive-displacement pipette. Forty-eight hours post-PsV challenge, mice were vaginally instilled with 0.4 mg of 5′-F-Luciferin (CellCyto Life Sciences, China). Three minutes later, luciferase signals were acquired for 10 min with an IVIS 200 bioluminescent imaging system (Xenogen, USA).
